# Implications of the COVID-19 pandemic in monitoring health and cardiovascular diseases: survey study

**DOI:** 10.1590/1980-220X-REEUSP-2022-0112en

**Published:** 2023-06-19

**Authors:** Juliana Pereira Machado, Bárbara Caroliny Pereira Costa, Mayara Rocha Siqueira Sudré, Isabela Gomes Musa dos Santos, Eugenia Velludo Veiga

**Affiliations:** 1 Centro Universitário Barão de Mauá, Departamento de Enfermagem, Ribeirão Preto, SP, Brazil.; 2 Universidade de São Paulo, Escola de Enfermagem de Ribeirão Preto, Programa de Enfermagem Fundamental, Ribeirão Preto, SP, Brazil.; 3 Universidade de São Paulo, Escola de Enfermagem de Ribeirão Preto, Departamento de Enfermagem Geral e Especializada, Ribeirão Preto, SP, Brazil.

**Keywords:** COVID-19, Cardiovascular Diseases, Comprehensive Health Care, Pandemics, COVID-19, Enfermedades Cardiovasculares, Atención Integral de Salud, Pandemias, COVID-19, Doenças Cardiovasculares, Assistência Integral à Saúde, Pandemias

## Abstract

**Objective:**

To analyze the implications of COVID-19 in the monitoring of health and cardiovascular diseases in health services.

**Method:**

This is a descriptive, cross-sectional, survey-type study with 798 adults, selected by snowball on social networks, between June and July 2020. Data were collected in an electronic form validated for this study.

**Results:**

There was a negative impact on monitoring health and cardiovascular diseases with missed appointments and elective exams. Symptoms such as chest pain and hypertensive crisis were neglected due to fear of contagion, lack of knowledge or structural lack of services, in addition to impaired monitoring of chronic conditions.

**Conclusion:**

The severity of results is noted considering the COVID-19 progression and the risk of complications. It is necessary to organize flows and structures compatible with each care profile, in health services, to guarantee care and promote diagnosis and control of chronic conditions in the set of actions to contain pandemics. It is crucial to prioritize primary care in health follow-ups during pandemic periods, as this has a direct impact on the progression of critical conditions at other levels of care.

## RESUMO

**Objetivo:**

Analisar as implicações da COVID-19 no acompanhamento de saúde e doenças cardiovasculares em serviços de saúde.

**Método:**

Estudo descritivo, transversal, tipo *survey,* com 798 adultos, selecionados em bola de neve em redes sociais, entre junho e julho de 2020. Dados foram coletados em formulário eletrônico validado para este estudo.

**Resultados:**

Houve impacto negativo no acompanhamento de saúde e doenças cardiovasculares com perda de consultas e exames eletivos. Sintomas como dor torácica e crise hipertensiva foram negligenciados por medo de contágio, desconhecimento ou carência estrutural dos serviços, além do prejuízo no acompanhamento de condições crônicas.

**Conclusão:**

Nota-se gravidade nos resultados frente à evolução da COVID-19 e risco de complicações. É necessário organizar fluxos e estruturas compatíveis com cada perfil assistencial, nos serviços de saúde, para garantir atendimento e promover diagnóstico e controle de condições crônicas no conjunto de ações de contenção de pandemias. É determinante priorizar a atenção primária nos acompanhamentos de saúde em períodos pandêmicos, pois isso impacta diretamente na evolução dos quadros críticos dos demais níveis de atenção.

## RESUMEN

**Objetivo:**

Analizar las implicaciones de la COVID-19 en el seguimiento de la salud y las enfermedades cardiovasculares en los servicios de salud.

**Método:**

Estudio descriptivo, transversal, encuesta, con 798 adultos, seleccionados en bola de nieve en las redes sociales, entre junio y julio de 2020. Los datos fueron recolectados en un formulario electrónico validado para este estudio.

**Resultados:**

Hubo un impacto negativo en el seguimiento de la salud y enfermedades cardiovasculares con citas perdidas y exámenes electivos. Síntomas como dolor torácico y crisis hipertensiva fueron desatendidas por miedo al contagio, desconocimiento o carencia estructural de servicios, además del deterioro del seguimiento de las condiciones crónicas.

**Conclusión:**

Hay gravedad en los resultados ante la evolución de la COVID-19 y el riesgo de complicaciones. Es necesario organizar flujos y estructuras compatibles con cada perfil de atención, en los servicios de salud, para garantizar la atención y promover el diagnóstico y control de las condiciones crónicas en el conjunto de acciones para la contención de pandemias. Es crucial priorizar la atención primaria en los seguimientos de salud durante los períodos de pandemia, ya que esto tiene un impacto directo en la evolución de las condiciones críticas en otros niveles de atención.

## INTRODUCTION

Cardiovascular diseases (CVD) are the main cause of death among adults and the elderly, and it is estimated that it represents about 17.9 million deaths worldwide. Inadequate diets, sedentary lifestyle, tobacco use and harmful use of alcohol are the most important behavioral risk factors for the development and worsening of these diseases^(1)^.

During the COVID-19 pandemic, CVD became even more worrying due to the deleterious association between these conditions^(2,3)^. In addition to this, changes in people’s daily routines, such as the reduction in physical exercise, increased consumption of cigarettes and alcoholic beverages, which led to the worsening of cases^(4)^. 

To reduce the spread and transmission of COVID-19, the World Health Organization (WHO) has proposed some measures for its control, including social distancing and isolation^(5)^. Such measures compromised access to health services, and with that, affected the continuity of care provided to individuals with CVD^(6)^. 

Services expanded intensive care beds to care for patients with COVID-19 and, in primary care, had to remodel their care during the period of social isolation, even canceling consultations and other services^(7)^ On the other hand, emergency care (EC) services abruptly became COVID poles and remodeled their flow, which contributed to making it difficult to provide care that was not related to the pandemic^(4)^. Diagnostic services saw their schedule plummet^(8)^ and tertiary hospitals almost completely lost elective surgical demand^(9)^ for a period.

In this context, CVD prevention and treatment services were affected by the COVID-19 pandemic, not only due to isolation, but also due to difficulty in managing health demands. There was reorganization or discontinuation of routine services with consequent interruption of assistance to people undergoing treatment for CVD, in addition to patients undergoing cancer treatment, among others^(10)^.

Moreover, it was found that individuals with CVD were the most affected by the severe forms of COVID-19 requiring hospitalization and intensive care^(11,12)^. In severe cases, low oxygen demand can lead to acute myocardial infarction, causing myocardial injury in the form of acute myocarditis or secondary injury^(13)^.

The reduction or even interruption of elective care in health services as well as changes in the flow of EC constituted a threat to the health of people living with CVD during the COVID-19 pandemic. Considering the above, the present study aimed to analyze the implications of the COVID-19 pandemic in monitoring health and CVD in health services. The analysis was extended to the restrictions imposed by the services on individual health behavior in the studied sample. 

## METHOD

### Study Design

This is a descriptive, cross-sectional survey study^(14)^, with a quantitative approach, guided by the Strengthening the Reporting of Observational Studies in Epidemiology (STROBE) recommendations.

### Population

This study included people residing in Brazil who use public or private health services, and who had access to the internet. According to the Internet Management Committee in Brazil (IMC), in a study published in 2019, this population represents about 126.9 million individuals aged 10 years or more connected daily to the internet in all regions of the national territory^(15)^.

### Inclusion Criteria

This survey included in its sample users of health services, over 18 years old, who use any of the three levels of care (primary, secondary or tertiary), from the public or private network, without distinction of gender and ethnicity, who voluntarily agreed to participate. People without access to the internet or social networks were excluded so that they could access and respond to the questionnaire, with limitations in reading and writing the Portuguese language, or without interlocutors with the digital medium who could help in the task of answering the questionnaire, or even Brazilians residing outside Brazil. 

### Sample

The sample composition was non-probabilistic^(14)^, obtained by using the snowball strategy^(16)^, which provides for the recruitment of its participants through an invitation triggered by virtual communication channels and social networks. Participants who made themselves available to participate in the research were asked to recommend new participants when receiving the questionnaire and to share it on their social networks.

The option for this methodological strategy met specific requirements of the pandemic, as it respected social isolation and quarantine measures, in addition to favoring participation by using a global means of communication (the internet). Selection saturation occurred at the end of 10 days, after the last invitations were sent and no longer produced valid responses^(16)^, and the final sample had 798 viable participants. 

### Data Collection

For data collection, a questionnaire was prepared using Google Docs^®^, in the area of editing questionnaires and generating spreadsheets called Google Forms^®^, available and freely accessible on the internet. Because it is fast and cheap, it avoids typing or transcription errors as it archives the raw data, entered by the participants themselves. The questionnaire was designed to be self-administered, with questions about participants’ sociodemographic characteristics, respecting proposed ethical issues. It contained specific questions with dichotomous answers, such as “yes or no”, or multiple choice, in order to facilitate understanding and avoid text and semantic interpretation biases. 

The questionnaire was submitted to content and appearance validation by judges before being sent to participants. This process refers to the assessment of how much a sample of items is representative of what is intended to be measured, representing the degree of relevance in which the content of an instrument reflects. 

Regarding judge selection, aspects related to proximity to the theme of CVD and accessibility to health systems, with research training (master’s/doctoral degree), were considered, except for those who were not available to participate. Content validity by experts should assess a given instrument in relation to the significance, relevance and representativeness of its content and how much it is able to address the proposed topic^(17)^. Five professionals made up the sample of judges. The invitation to participate was given by email, using remote access tools (Google Forms^®^) to fill in the online form, complying with current ethical precepts. 

The judges assessed each question in terms of content, meeting the proposed objectives and statement semantics. All assessments were favorable to content, and there were some adjustments to the statement in order to favor understanding at the time of data collection, as it is self-applicable. The suggested corrections were accepted, and the questionnaire structure was adjusted. 

The final version of the questionnaire had 40 questions related to sociodemographic characteristics, such as health history, past illnesses, CVD being treated (hypertension, cardiac arrhythmia, heart failure, coronary artery disease, angina, stroke), main conditions associated with CVD, such as kidney disease, diabetes, hypercholesterolemia, current health treatments. Moreover, it investigated signs and symptoms manifested during the period studied and attendances at health services during the pandemic. 

Subsequently, a pre-test was carried out to validate the questionnaire, with 10 users of health services randomly chosen through social networks, respecting ethical precepts and with the same approach offered to the study participants, but they did not participate in the final sample. A pilot test had positive feedback, and participants did not report difficulties. 

Once the final version of the questionnaire was tested, it was published on Facebook^®^, WhatsApp^®^, Instagram^®^ and e-mail, containing a presentation of the study and its objectives, in order to recruit eligible participants from June 16, 2020, under daily monitoring of responses until July 1, 2020, when the last valid response was sent. After a week without new responses, the collection ended on July 07, 2020. Access to data from these potential participants was obtained through the first wave^(14)^ of invitations, originating from members of the research group to which the researchers are linked. Upon receiving the invitation, people were instructed to reply to their respective contacts and so on, forming waves of invitations with the generation of viable data^(18)^. 

Each participant had to access a link that led to the questionnaire. On the home page, next to the project presentation, access to the Informed Consent Form (ICF) was made available to participants, via Dropbox, which allowed participants to read and file this document.

Then, participants automatically accessed the available questions, to answer them and, when completing the filling, the answers were automatically saved in a specific database in the cloud and offline, through extension applications installed directly from Google^®^. The database was automatically saved, with secure and restricted access to the research team. Data collection took place in June and July 2020.

### Data Analysis and Treatment

Data were tabulated in an Excel spreadsheet imported from Google Docs^®^, and analyzed using the STATA^®^ 13 software. The study results were presented in descriptive statistics, containing frequencies, percentages and standard deviation for all variables studied. To assess the existence of an association between the occurrence of the symptom and seeking care, Pearson’s chi-square and Fisher’s exact tests were used, with a significance of 0.05. All analyzes were performed using the R program.

### Ethical Aspects

The study was approved by the Research Ethics Committee of the *Escola de Enfermagem de Ribeirão Preto*, *Universidade de São Paulo*, under Opinion 4.039.506/2020, in line with Resolution 466/12 on ethics in research with human beings.

## RESULTS

In order to analyze the implications of the COVID-19 pandemic in monitoring health and CVD in health services, from the point of view of their users, this study has a sample of 798 participants between 18 and 85 years old (mean 40.6, median 39 and SD= 14.4), mostly female (74.1%), aged between 21 and 50 years (69.8%), only 121 (15.2%) between 51 and 59 years old and only 87 people (10.9%) aged 60 years or older (Table 1). 

**Table 1:** Sociodemographic characteristics (n = 798) – Ribeirão ­Preto, SP, Brazil, 2020.

Sociodemographiccharacteristics		
Age in full years	n	%
18–20	33	4.1
21–23	68	8.5
24–26	53	6.6
27–29	70	8.8
30–32	57	7.1
33–35	58	7.3
36–38	47	5.9
39–41	56	7.0
42–44	47	5.9
45–47	46	5.8
48–50	55	6.9
51–53	28	3.5
54–56	53	6.6
57–59	40	5.0
60–62	28	3.5
63–65	20	2.5
66–68	13	1.6
69–71	11	1.4
72–74	7	0.9
75–77	2	0.3
78–80	4	0.5
81–85	2	0.3
**Complete education**		
Elementary school	17	1.8
High/vocational school	135	16.9
Higher education	312	39.1
Graduate degree	334	41.9
**Marital status**		
Married/stable/cohabiting partner	467	58.5
Single	255	31.9
Divorced or separated/widowed	76	9.6
General total	798	100.0

Source: the authors.

Among the participants, 334 (41.9%) have a graduate degree; 467 (58.5%) have a marital relationship; and 459 (57.5%) live with two or three people. As an address, the state of São Paulo predominates, with 631 (79.1%) participants, followed by Minas Gerais, with 78 (9.8%) and Mato Grosso, with 30 (3.8%), Paraná and Rio de Janeiro, with 13 (1.6%) and 9 (1.1%), respectively, and the remainder is distributed in the other Brazilian states.

Regarding the profile of use of health services, of the total number of participants, 569 (71.3%) have a health insurance and report using this network when seeking care in consultations, EC or hospitalizations, and 100 (12.5%) are monitored by the Unified Health System (SUS – *Sistema* Único *de Saúde*) in Basic Health Units (BHU), specialty outpatient clinics or Family Health Strategies (FHS) and in the hospital network. Only eight (1.0%) have private follow-up for appointments or hospital admissions. In the period studied, there are 118 (14.8%) reports of people who do not undergo medical follow-up.

This study sought to survey ongoing health treatments during the first half of the pandemic in the context of CVD and other altered health conditions treated in the period. Specifically, regarding previous cardiovascular events, there are reports of acute myocardial infarction (1.6%) and stroke (1.1%) in the sample. 

Among the 798 participants, this survey shows 324 (40.6%) reports of at least one ongoing health treatment related to CVD, in addition to diabetes and kidney disease. The other participants (59.4%) stated that they were not undergoing health treatment during the collection period and sought health services only in emergency cases. Still, 104 participants reported having a health problem (self-reported) but not treating it, among which asthma and arthritis. Hypertension appears in 182 (56.2%) of CVD reports, followed by dyslipidemia (55/17.0%). Other people also mention that they treat several conditions associated with CVD, here called heart disease, as shown in Table 2. 

**Table 2:** Cardiovascular disease treatments ongoing in the pandemic (n = 324) – Ribeirão Preto, SP, Brazil, 2020.

Cardiovascular disease treatment	n	%
Hypertension	182	56.2
Dyslipidemia	55	17.0
Diabetes	45	13.9
Heart disease*	12	3.7
Kidney disease	5	1.5
Heart disease* + hypertension	7	2.2
Heart disease* + hypertension + dyslipidemia	6	1.9
Heart disease* + hypertension + diabetes	7	2.2
Heart disease* + dyslipidemia	2	0.6
Heart disease* + diabetes	1	0.3
Heart disease* + diabetes + kidney disease	2	0.6
TOTAL	324	100.0

*Heart disease (heart failure, arrhythmia, or other heart problem). Source: the authors.

The data obtained also show 216 (27.1%) reports of other health treatment in progress, among all participants (798/100%), of which 62 (28.7%) conditions related to psychiatric disorders stand out. Table 3 explores other ongoing treatments. 

**Table 3:** Other self-reported health treatments currently ongoing (n = 216) – Ribeirão Preto, SP, Brazil, 2020.

Other health treatments	n	%
Psychiatric (depression/anxiety/panic/binge eating/sleep disorder)	62	28.7
Endocrinological (hypothyroidism/obesity/post bariatric)	45	20.8
Dermatological	16	7.4
Rheumatologic (arthritis/arthrosis/osteoporosis)	15	6.9
Pulmonological (asthma/bronchitis/rhinitis)	13	6.0
Gynecological (menopause/polycystic ovary/endometriosis)	12	5.6
Gastroenterological (gastritis/reflux/Crohn’s)	11	5.1
Neurological (migraine/seizures/stroke)	8	3.7
Ophthalmological	7	3.2
Renal	5	2.3
Oncological	5	2.3
Obstetric	3	1.4
Urological	2	0.9
Hematologic (thrombosis/thrombocytopenia)		0.9
Orthopedic	2	0.9
Others*	8	3.7
Total	216	(100.0)

* Physiotherapy service, kidney and liver transplantation, infectologist, nutritionist. Sources: the authors.

This study also questioned the problems or altered health conditions that were not treated in the investigated period, and the data reveal 119 (14.9%) people in this condition. As a highlight in this quantitative, 27 (12.3%) respiratory ­problems, 21 (9.6%) endocrine and 11 (5.0%) gastrointestinal ­disorders appear.

 Regarding medical appointments scheduled for the first half of 2020, 355 (44.5%) of participants reported some appointment. However, approximately one third (126) were not performed due to cancellation by the service or patients chose not to attend. 

Likewise, 270 (33.8%) participants had at least one scheduled elective exam. A total of 191 (70.7%) were performed, while 79 (29.2%) were canceled or postponed by the service, or the patient did not attend. Among these tests that were not performed, imaging and cardiac tests were more affected, including magnetic resonance imaging, computed tomography scans, echocardiograms, exercise tests, among others, as shown in Table 4. 

**Table 4:** Medical tests not performed (n = 79) – Ribeirão Preto, SP, Brazil, 2020.

Type of tests	Cancelled		Rescheduled		Patient did not attend	
	n %		n %		n %	
Imaging tests	12	(15.2)	2	(2.5)	6	(7.6)
Cardiological tests	3	(3.8)	3	(3.8)	9	(11.4)
Blood tests	3	(3.8)	6	(7.6)	7	(8.9)
Gynecological tests	3	(3.8)	6	(7.6)		
Endoscopy/colonoscopy	3	(3.8)	6	(7.6)		
Vision test	4	(5.1)	0	(0.0)		
Biopsy	2	(2.5)	1	(1.3)		
Others	2	(2.5)	1	(1.3)		
Total	32		25		22	

Source: the authors.

This study questioned whether the participant sought a health service between March and July 2020, whether in scheduled situations or not, and the data found reveal 346 (43.3%) patients who had some type of care. Of these, 158 (45.7%) are consultations that took place in an office. According to the data collected, regarding the type of health care provided, hospital care (84/24.3%) or Emergency Care Units (ECU) (36/10.4%) stand out. There are also reports of 36 (9.8%) in BHU and only 28 (8.1%) via telemedicine, with consultations mediated by remote access or video calls by messaging applications. 

According to the results, 342 (42.9%) participants reported a complaint or some symptom since the beginning of the pandemic until July 2020, including fever, renal colic and pain, including chest pain. Of these symptoms, most (282/82.4%) contain reports potentially related to CVD, such as chest pain and chest pain that radiates to the neck, in addition to palpitations or arrhythmias. More than half of these people did not seek care and may have neglected a serious cardiovascular clinical manifestation, especially pain in the thoracic region and hypertensive crisis, with a statistical association (p < 0.05), as shown in Table 5. 

**Table 5:** Cardiovascular symptoms (n = 282) reported by participants during the period between March and July 2020 – Ribeirão Preto, SP, Brazil, 2020.

Reported symptom	Did not seek care (561)	Sought care (237)	p-value
	n %	n %	
Chest pain	33 (5.9)	24 (10.1)	0.0330*
Chest pain that radiates to the neck	27 (4.8)	16 (6.8)	0.2680*
Back pain in the chest and lung region	22 (3.9)	21 (8.9)	0.0050*
Palpitations (arrhythmias)	33 (5.9)	21 (8.9)	0.1260*
Tingling in the limbs	25 (4.5)	12 (5.1)	0.7090*
Dizziness	17 (3)	9 (3.8)	0.5770*
Leg weakness	4 (0.7)	5 (2.1)	0.1350**
Fainting feeling	1 (0.2)	0 (0)	1.0000**
High blood pressure crisis	3 (0.5)	9 (3.8)	0.0010**

Pearson’s chi-square test* and

Fisher’s exact test**. Source: the authors.

According to the results presented, 60 people had chest pain and did not seek the health service. Symptoms of chest pain (0.033), back pain in the chest and lung region (0.005) and hypertensive crisis (0.001), according to the tests performed, are associated with the demand for the health service, something extremely relevant in times of a pandemic, considering the many changes in location and flow of care in emergency services. 

When these cardiovascular symptoms are analyzed among the 324 patients with CVD, the findings are even more worrying, as 49 (15.1%) people reported chest pain with or without irradiation to the arm with potential clinical relevance and did not seek care, which may pose a high health risk. These results show important symptoms being neglected by people undergoing treatment for CVD. 

## DISCUSSION

This study analyzed the implications of the COVID-19 pandemic on health and CVD monitoring in health services and the results showed changes in elective care and also in emergency care. Data revealed cancellation of consultations and elective exams, with potential impact on the various health treatments in progress, especially in CVD. Also, important cardiovascular symptoms such as chest pain, thoracic pain and hypertensive crisis were neglected since they did not seek the health service.

Data analysis showed a certain peculiarity in relation to the general Brazilian population, as young adult participants, married, and with income ranging from four to six minimum wages prevailed. This may be related to the profile of internet users and social networks, given that the approach provided for in the method took place in the digital environment. 

This study brought mostly graduate participants, which differs from national data. The level of education for higher education estimated for people aged 25 or over in Brazil in 2019 was 17.4%^(18)^, i.e., a lower value than the data found in this study. In this sense, considering that the snowball selection model tends to obtain new participants with a similar profile, due to the way selection is carried out^(17)^, it is possible to understand that most participants have a graduate degree, considering that the beginning of sample selection took place in the academic environment and, from there, in their contact networks. 

Another result that remains divergent from the Brazilian population in general refers to the average income of the participants. The level of education has been shown to be associated with the average income of Brazilians, and people with complete higher education have, on average, an income almost six times higher than that of workers without education^(18)^. This data corroborates those found in this study, considering the levels of education above the national average.

As for the means of access to health, there was a predominance of participants with private insurance. According to the Brazilian National Supplementary Health Agency (ANS - *Agência Nacional de Saúde Suplementar*)^(19)^, medical-hospital health insurances registered, between April 2020 and April 2021, an increase of 1.05 million in the total number of users in the country, mainly in the states of São Paulo, Minas Gerais and Goiás, which converges with the results obtained. 

Regarding the studied population’s health history, there was a high prevalence of hypertension, which is a cause for concern in times of a pandemic, as it requires specific and continuous care. After all, people with CVD may have more complications with SARS-CoV-2 infection and require strict and systematic follow-up^(20)^. 

Literature review carried out on CVD and its relationship with COVID-19 reaffirms the importance of this follow-up, even in periods of isolation, and suggests attention regarding the behavior of these patients in continuity of their treatment. In addition to this, it questions how much the pandemic has impacted the population’s access to health services, leading to decompensation of the disease^(21)^.

Another relevant aspect in this survey refers to the psychiatric alterations mentioned by the participants: 28.7% of the ongoing treatments during the studied period (Table 3) that can influence the occurrence or worsening of CVD, especially anxiety disorders, binge eating and depression. In our midst, much has been said in the media about the damage to mental health in the general population during the pandemic. A recent study raised data on weaknesses in social, emotional and psychological aspects resulting from the pandemic. Periods of isolation, fear of contagion and social crises, in a broad sense, can strongly contribute to the occurrence of disorders such as depression and anxiety^(22)^. 

In the context of social isolation, there is a decrease in access to health services for this population, and psychological interventions have been carried out by remote access, via the internet, telephones and letters, in order to also contain this damage caused by COVID-19. Such mechanisms made it possible to accept complaints related to mental health^(23)^. 

This survey revealed other health treatments in the sample studied, with emphasis on endocrine treatments, such as dyslipidemia, hypothyroidism and obesity, with high relevance when associated with CVD, with 20.8% of ongoing treatments in the period (Table 3). Endocrine diseases are also considered chronic and are present in a large part of the global population, which makes them a priority target for prevention and control^(1)^.

In the studied sample, of the scheduled medical appointments, about a third were not carried out because the service canceled or the patient gave up. There is an alert here for losses in health follow-ups. In the context of CVD, one can imagine how much these cancellations effectively contributed to decompensate clinical conditions and how much they contributed to the complications of COVID-19. In fact, studies have shown concern about the impact of COVID-19 in relation to cardiovascular comorbidities, and have revealed the severity and lethality of CVD when associated with SARS-CoV-2^(24,25)^. 

Regarding the high prevalence of hypertension in our sample, a Chinese study corroborates this profile, identifying hypertension with the highest prevalence among participants^(26)^, and reinforces that these are not considered a risk factor for infection by COVID-19. However, hypertension confers a greater risk of complications from the disease^(25)^.

Among the elective exams, there was a significant loss of exams due to the cancellation of the service, mostly due to patient withdrawal. Similar data of this behavior were evidenced in Italian health services, in the same period, when 32.4% faced a delay of a scheduled medical service and 13.2% refused to go to the office for fear of contracting COVID-19^(27)^. 

In general, this study revealed a decrease in imaging exams of around 25%, between cancellations, rescheduling and patient absence. This impact was recorded with even greater proportions in a North American study, in which system-wide imaging volume decreased by 55% in April 2020, with mammograms and nuclear medicine exams being the most affected modalities, decreasing by 93% and 61%, respectively^(9)^. 

Faced with the loss of elementary screening tests and the postponement of control tests, new considerations arise about the deleterious impact of the pandemic on the monitoring of chronic conditions, with special alert to the areas of cardiology and oncology. Nowadays, when a large part of the population is fully or partially immunized, and the ICU maintains a stable operational capacity, it is suggested a movement to rescue and attract patients who have stopped undergoing outpatient health follow-up. 

Oncological diseases are considered a public health problem and are co-responsible for changing the disease profile of the population worldwide^(28)^. As with CVD, screening for colorectal and breast cancer prevention, both with imaging tests, should be performed annually from the age of 40^(28)^. 

Alarming results revealed, in this sample, that potentially severe cardiovascular symptoms were not assessed in health services (Table 5). Sixty participants reported precordial pain with and without irradiation, and another 25 had dizziness, tingling in their hands, among others, and did not seek health care. One reason to be considered could be fear of contagion as well as lack of information regarding changes in care flows in many services, considering that, on the period studied, did not yet have a vaccine in our midst. Moreover, many changes in the flow of care were necessary to meet the exponential demand for COVID-19 cases. 

It is evident that, in a way, not all are in fact cardiovascular events, but there is an undeniable risk of having a serious event and not seeking care. These results confirm another Brazilian study in which people reported avoiding attendance at health services, even knowing that it could be something more serious^(29)^. 

If there was negligence in seeking care in the face of a considerable symptom, this study revealed a significant association between potentially critical symptoms (chest pain and hypertensive crisis) and seeking care. If the care networks are constantly focused on educating the population to seek a service in the face of certain symptoms, as the time factor is crucial in the life of a person with an acute myocardial infarction or stroke, among others, it is expected that the population will seek care in the looming symptoms such as chest pain. The concern, in this sense, is precisely the effective maintenance of lines of care for acute clinical, surgical and obstetric conditions in health services, concomitant with pandemic scenarios. 

This is, therefore, a new global challenge for health services, since, in addition to the need to structure themselves internally and in their care networks to deal with the COVID-19 pandemic, which is not over yet, there is a need to strongly consider strategies to keep emergency services active as well as surgical and obstetric centers as well as fully reopening outpatient services as soon as possible to care for patients, so as to reduce the risk of delayed diagnosis and treatment.

In this regard, there are urgent demands on how to guarantee protection against the spread of infections among patients and also among professionals. For this, strategies are needed to maximize quality, reduce the repetition of procedures, improve patient experience while reducing the risk of infection for staff and ensuring sufficient capacity, with technical and operational innovation, decreased length of stay rates and technical skill^(30)^.

Limitations of this study are related to the methodological characteristics of the snowball sample selection, used to comply with sanitary requirements of social distancing, which make the sample potentially distorted, considering that it emerges from a specific group and tends to maintain the profile according to the contact networks of those involved. Thus, there is no way to establish a comparison between regions, nor to generalize results. For this, it is suggested to carry out further studies.

Results of this survey contribute to advances in nursing/health by evidencing losses in the monitoring of chronic conditions, as they give the services opportunities for review and operational structuring that minimize the damage caused by this pandemic, and prepare them for other periods of health crisis. 

## CONCLUSION

By analyzing the implications of the COVID-19 pandemic on health and CVD monitoring in health services, this study obtained evidence that suggests a negative impact on interrupted or postponed exams and consultations, but above all in complaints potentially related to cardiac conditions, in which people neglected important symptoms, such as chest pain. The results showed that the pandemic had an impact on the postponement of consultations and tests as well as on not looking for the health service even in the imminence of symptoms considered important, in the studied sample. These results comprise scientific evidence that confirm data reported by the media on the reduction of routine demands on health services. 

The severity of results is noted as COVID-19 progression is analyzed, considering the brutal involvement of this infection among people with comorbidities associated with CVD. Furthermore, at the same time, there is concern about the deleterious association between COVID-19 and CVD, as the neglected symptoms reveal the risk of a serious cardiovascular event, many of which go unattended due to fear of contagion or changes in care at the height of the pandemic. 

It is evident, therefore, the need to organize flows and structures compatible with each care profile, at different levels and health services, in order to guarantee and maintain elective and emergency care, promote diagnosis and follow-up of chronic conditions, as part of of a set of actions to contain pandemics. In other words, it is predictive that primary care is prioritized in the promotion and maintenance of health in pandemic periods, as this directly impacts the progression of critical conditions at other levels and care. Moreover, the secondary and tertiary levels must be prepared to optimize and guarantee the fulfillment of their demand in different flows in times of pandemic.
